# Risk stratification based on acute-on-chronic liver failure in cirrhotic patients hospitalized for acute variceal bleeding

**DOI:** 10.1186/s12876-023-02768-6

**Published:** 2023-05-12

**Authors:** Zongyi Zhu, Huiqing Jiang

**Affiliations:** 1 Department of Gastroenterology, the Second Hospital of Hebei Medical University; Hebei Key Laboratory of Gastroenterology; Hebei Institute of Gastroenterology, Hebei Clinical Research Center for Digestive Diseases, Shijiazhuang, Hebei China; 2 Department of Gastroenterology, Weixian People’s Hospital, Xingtai, Hebei China

**Keywords:** Acute variceal bleeding, Acute-on-chronic liver failure, Risk stratification, Prognosis

## Abstract

**Background and aims:**

Acute variceal bleeding (AVB) is a life-threatening complication of cirrhosis. Acute-on-chronic liver failure (ACLF) is a syndrome characterized by acute decompensation of cirrhosis, multiple organ failures and high short-term mortality. This study aimed to evaluate the role of ACLF in the risk stratification of cirrhotic patients with AVB.

**Methods:**

Prospective data of 335 cirrhotic patients hospitalized for AVB were retrospectively extracted from Medical Information Mart for Intensive Care (MIMIC)-IV database. ACLF was defined by European Association for the Study of Liver-Chronic Liver Failure Consortium and diagnosed/graded with chronic liver failure-organ failure (CLIF-OF) score. Cox-proportional hazards regression analysis was performed to identify the risk factors for 6-week morality in AVB patients. Discrimination and calibration of prognostic scores were evaluated by plotting the receiver operating characteristics (ROC) curve and calibration curve, respectively. Overall performance was assessed by calculating the Brier score and R^2^ value.

**Results:**

A total of 181 (54.0%) patients were diagnosed with ACLF (grade 1: 18.2%, grade 2: 33.7%, grade 3: 48.1%) at admission. The 6-week mortality in patients with ACLF was significantly higher than that in patients without ACLF (43.6% *vs.* 8.4%, *P* < 0.001) and increased in line with the severity of ACLF (22.5%, 34.2% and 63.8% for ACLF grade 1, 2 and 3, *P* < 0.001). In multivariate analysis, presence of ACLF remained as an independent risk factor for 6-week mortality after adjusting for confounding factors (HR = 2.12, *P* = 0.03). The discrimination, calibration and overall performance of CLIF-C ACLF and CLIF-C AD were superior to the traditional prognostic scores (CTP, MELD and MELD-Na) in the prediction of 6-week mortality of patients with and without ACLF, respectively.

**Conclusion:**

The prognosis of cirrhotic patients with AVB is poor when accompanied by ACLF. ACLF at admission is an independent predictor for the 6-week mortality in cirrhotic patients with AVB. CLIF-C ACLF and CLIF-C AD are the best prognostic scores in AVB patients with and without ACLF, respectively, and can be used for the risk stratification of these two distinct entities.

## Introduction

Acute variceal bleeding (AVB) is one of the most common and serious complications of cirrhosis. Despite recent improvement in therapy (medication, endoscopy), up to 10–15% of patients still have persistent bleeding or early rebleeding and the overall mortality with each episode of AVB is still approximately 15% to 25% at six weeks [1–3]. Early identification of these high-risk patients and implementation of alternative more effective treatments, such as preemptive transjugular intrahepatic portosystemic shunt (p-TIPS), were recommended by multiple international consensus to improve the prognosis of this entity [4–6]. So far, factors found to be associated with the poor prognosis of cirrhotic patients with AVB include shock on admission, hepatic venous pressure gradient (HVPG) > 20 mmHg, concurrence of hepatic encephalopathy (HE) or hepatocellular carcinoma (HCC), renal failure and bacterial infection, etc. [7, 8]. Some scoring systems, such as Child-Turcotte-Pugh (CTP) [9], Model for End-stage Liver Disease (MELD) [10] and MELD-Na [11] have been proposed for predicting the prognosis of these patients. However, as these scores mainly reflect the severity of liver disease, their prediction accuracy may decrease when they encounter extrahepatic organ failures which have a vital impact on the prognosis of these patients [12, 13].

Acute-on-chronic liver failure (ACLF) was first defined by European Association for the Study of Liver-Chronic Liver Failure Consortium (EASL-CLIF) in the CANONIC study [14] as a syndrome characterized by acute decompensation (ie, ascites, hepatic encephalopathy, AVB and bacterial infection) of cirrhosis, multiple organ failure and high short-term mortality. In CANONIC study, the 28-day mortality in cirrhotic patients with ACLF is significantly higher than that in those without ACLF (33.9% *vs.* 4.7%, *P* < 0.001). Nowadays, with the progress of hemostasis technology, the proportion of AVB patients dying of hemorrhagic shock is gradually decreasing. Instead, most patients died of liver failure or multiple organ failures [14]. Although AVB is a well recognized precipitant leading to the occurrence and development of ACLF [15–17], the role of ACLF in the prognosis of cirrhotic patients with AVB has not yet been fully investigated.

In this study, we aimed at addressing the following 3 clinically relevant issues: (1) Whether the presence and grade of ACLF at admission was independently associated with the poor prognosis of cirrhotic patients hospitalized for AVB. (2) To analyse the difference of demographic characteristics, clinical features and laboratory parameters between AVB patients with and without ACLF (mere acute decompensation, AD). (3) To identify the accurate and robust prognostic scores in patients with ACLF and AD, respectively.

## Patients and methods

### Patients

Medical data of patients in this retrospective cohort study was obtained from Medical Information Mart for Intensive Care (MIMIC)-IV (version 2.0) [18, 19], which is a large and freely-available database comprising anonymous medical data of patients admitted to the intensive care units of Beth Israel Deaconess Medical Center between 2008 and 2019. Inclusion criteria: cirrhotic patients hospitalized for AVB. Exclusion criteria: (1) Less than 18 years old. (2) Pregnancy. (3) Without ICU stays. (4) Incomplete records. (5) HCC or malignant tumor in other organs. (6) Human immunodeficiency virus (HIV) infection or ongoing immunosuppressive therapy. (7) Serious extrahepatic diseases. Access to MIMIC-IV database was approved by the Institutional Review Board of the Beth Israel Deaconess Medical Center and Massachusetts Institute of Technology after the completion of online course and examination. Given the public availability of MIMIC-IV in which all patients' private information is anonymous, the approval by local ethics committee was waived.

### Data collection

Medical data of cirrhotic patients hospitalized for AVB in MIMIC-IV 2.0 database was extracted through Postgres Structured Query Language (PostgreSQL) programming in Navicat Premium (version 15.0.12), including age, gender, race, first day vital signs, mean arterial pressure, percutaneous oxygen saturation, consciousness score, first day laboratory parameters, vasopressors therapy, mechanical ventilation therapy, renal replacement treatment (RRT) therapy, severity scores and survival information, etc. For those admitted multiple times to ICU, data from their first admission was used. For laboratory indicators measured more than one time, we selected the maximum or minimum of them according to their clinical implications, such as the maximum of bilirubin or minimum of albumin.

### Calculation of prognostic scores

For all included patients, we calculated the traditional prognostic scores (CTP, MELD and MELD-Na). CTP score [9] was calculated as described previously. MELD score was directly obtained from MIMIC-IV. MELD-Na score was calculated using formula of: MELD-Na = MELD + 1.59 (135-Na), with maximum and minimum Na of 135 and 120 mEq/L, respectively [11]. In addition, we calculated the CLIF-C ACLF and CLIF-C AD score for patients with ACLF and AD, respectively, considering their corresponding specificity for the two distinct entities. CLIF-C ACLF score was calculated using formula of: CLIF-C ACLF = 10 × [0.33 × CLIF-OF score + 0.04 × Age (years) + 0.63 × Ln(WBC{10^9^cells/L}) − 2] [20]. CLIF-C AD score was calculated using formula of: CLIF-C AD = 10 × [0.03 × Age(years) + 0.66 × Ln(Creatinine{mg/dL}) + 1.71 × Ln(INR) + 0.88 × Ln(WBC{10^9^cells/L}) − 0.05 × Sodium(mmol/L) + 8] [21]. CLIF-OF score [20] was calculated as described previously.

### Diagnostic criteria

Ascites, hepatic encephalopathy and bacterial infection were defined and graded according to International Ascites Club [22], West Haven Criteria [23] and international guidelines [24, 25], respectively. ACLF was defined by EASL-CLIF and diagnosed/graded according to CLIF-OF score. Specifically, liver failure was defined by total bilirubin ≥ 12 mg/dl; coagulation failure was defined by INR ≥ 2.5; kidney failure was defined by creatinine > 2 mg/dL or requirement of RRT; circulatory failure was defined by requirement of vasopressor therapy to maintain blood pressure; respiratory failure was defined by PaO2/FiO2 ≤ 200 or SpO2/FiO2 ≤ 214 or requirement of mechanical ventilation for reasons other than airway protection and in the absence of HE grade III or IV; brain failure was defined by HE grade III or IV (West Haven). Grade 1 ACLF was defined as: (1) single kidney failure; (2) single failure of the liver, coagulation, circulation or respiration along with a serum creatinine level ranging from 1.5 to 1.9 mg/dL and/or mild to moderate HE; (3) single cerebral failure along with a serum creatinine level ranging from 1.5 and 1.9 mg/dL. Grade 2 and 3 ACLF were defined as having 2 and ≥ 3 organ failures, respectively.

### Study outcomes

The outcome of this study was 6-week all-cause mortality according to the Baveno VI Consensus Workshop [3]. Follow-up began on the date of patient’s admission and ended at 6 weeks later or the date of patient’s death.

### Statistical analysis

Variables with normal or skewed distribution based on the result in Kolmogorov–Smirnov test (*P* > 0.05 for normal distribution) were described as means (± standard deviation) and medians (interquartile range), respectively. Comparisons of normal and skewed distribution variables between groups were performed with independent sample t-test and Mann–Whitney test, respectively. Categorical variables were described as numbers (percentage) and compared with Chi-square test. Univariate and multivariate Cox-proportional hazards regression analysis were performed to identify the risk factors for 6-week morality in AVB patients. Variables with *P* value < 0.05 in univariate analysis were considered for multivariate analysis with backward stepwise method. To evaluate the performance of prognostic scores in predicting 6-week mortality, discrimination, calibration and overall performance of each score were evaluated. Discrimination refers to the ability of a prediction score in stratifying patients according to their risk of developing the outcome. Calibration refers to the ability of predicting absolute risks (how closely the predicted probabilities agree with the actual outcomes). Discrimination was evaluated by plotting receiver operating characteristics (ROC) curve and calculating the area under curve (AUC) and compared by DeLong test. Calibration was evaluated by performing Hosmer–Lemeshow goodness-of-fit test and plotting the calibration curve to visually observe the consistency between predicted and actual mortality. Overall performance was assessed by calculating the Brier score and R^2^ value, a lower Brier score or higher R^2^ value indicating a better overall performance. Cumulative survival curves were plotted with Kaplan–Meier method and compared with log-rank test. Statistical analysis and figure plotting were performed using MedCalc 19.0.4, STATA 15.0 and R 4.2.0. A two-tailed *P* value < 0 0.05 was considered to be statistically significant.

## Results

### Baseline characteristics

A total of 603 consecutive patients with AVB were screened, and 268 patients were excluded for the following reasons: without cirrhosis (*n* = 46), without ICU stays (*n* = 129), incomplete records (*n* = 11), malignant tumor including HCC (*n* = 62), HIV infection (*n* = 13) and serious extrahepatic diseases (*n* = 7). Finally, 335 patients with cirrhosis and AVB who met the inclusion and exclusion criteria were included in this study. Their baseline characteristics were shown in Table 1. Patients were predominantly male (68.4%) and white (63.6%), with a median age of 55 years. The main etiology of cirrhosis was alcohol (60.0%). A total of 200 (59.7%), 201 (60.0%), 98 (29.2%) and 35 (10.4%) patients had ascites, hepatic encephalopathy, bacterial infection and portal vein thrombosis at admission, respectively. 181 (54.0%) patients had ACLF at admission (18.2%, 33.7% and 48.1% for grade 1, grade 2 and grade 3, respectively). The baseline characteristics of patients with and without ACLF were similar in terms of race, age, gender and etiology of cirrhosis. As expected, ACLF patients more frequently presented with bacterial infections and had significantly higher inflammatory markers such as peripheral white blood cell (WBC) count than AD patients. In addition, ACLF patients had higher level of total bilirubin, alanine aminotransferase, aspartate aminotransferase, international normalized ratio, prothrombin time, creatinine, blood urea nitrogen, potassium and lower level of hemoglobin, albumin and serum sodium at baseline. The clinical conditions of ACLF patients seems much more severe than that of AD patients, such as the more instability of vital signs (faster heart rate, lower mean arterial pressure and ratio of percutaneous oxygen saturation to fraction of inspired oxygen), higher proportion of ascites/hepatic encephalopathy and worse prognostic scores (CTP, MELD, MELD-Na) than AD patients. Besides, length of ICU stay and hospital stay in ACLF patients were significantly higher than that in AD patients. A total of 5 (1.5%) patients [AD = 1 (0.6%), ACLF = 4 (2.2%), *P* = 0.24] received liver transplantation.

**Table 1:** Baseline characteristics of all patients and patients with or without ACLF (*N* = 335)

Baseline characteristics	All patients (*n* = 335)	AD (*n* = 154)	ACLF (*n* = 181)	*P* value
Age (years)	55.3 ± 11.9	55.7 ± 12.1	54.9 ± 11.7	0.50
**Gender n (%)**				0.31
Male	229 (68.4)	101 (65.6)	128 (70.7)	
Female	106 (31.6)	53 (34.4)	53 (29.3)	
**Race n (%)**				0.06
White	213 (63.6)	106 (68.8)	107 (59.1)	
Black	23 (6.9)	7 (4.5)	16 (8.8)	
Hispanic/Latino	22 (6.6)	14 (9.1)	8 (4.4)	
Asian	4 (1.2)	2 (1.3)	2 (1.1)	
Others	13 (3.9)	5 (3.2)	8 (4.4)	
Unknown	60 (17.9)	20 (13.0)	40 (22.1)	
**Etiology of cirrhosis n (%)**				0.19
Alcohol	201 (60.0)	84 (54.5)	117 (64.6)	
Virus	65 (19.4)	36 (23.4)	29 (16.0)	
Alcohol + Virus	21 (6.3)	12 (7.8)	9 (5.0)	
Nonspecific and others	48 (14.3)	22 (14.3)	26 (14.4)	
Liver transplantation n (%)	5 (1.5)	1 (0.6)	4 (2.2)	0.24
**Decompensation at admission n (%)**				
Ascites	200 (59.7)	63 (31.5)	137 (68.5)	** < 0.001**
Hepatic encephalopathy	201 (60.0)	58 (37.7)	143 (79.0)	** < 0.001**
I + II	107 (31.9)	42 (27.3)	65 (35.9)	
III + IV	94 (28.1)	16 (10.4)	78 (43.1)	
Bacterial infection	98 (29.3)	29 (18.8)	69 (38.1)	** < 0.001**
Pneumonia	36 (10.7)	13 (8.4)	23 (12.7)	
Urinary tract infection	31 (9.3)	8 (5.2)	23 (12.7)	
SBP	25 (7.5)	7 (4.5)	18 (9.9)	
Cholangitis	5 (1.5)	3 (1.9)	2 (1.1)	
Others	15 (4.5)	4 (2.6)	11 (6.1)	
Portal vein thrombosis	35 (10.4)	16 (10.4)	19 (10.5)	0.97
**Vital signs**				
Mean arterial pressure (mmHg)	75 (69–81)	79 (71–87)	72 (68–78)	** < 0.001**
Heart rate (bpm)	87 ± 17	84 ± 15	90 ± 17	**0.001**
SPO2/FIO2	448 (165–463)	456 (198–464)	246 (138–461)	**0.005**
**Laboratory tests**				
White blood cell (10^9^/L)	10.6 (6.8–16.3)	7.7 (5.4–11.3)	13.9 (9.7–20.8)	** < 0.001**
Hemoglobin (mg/dL)	8.1 (7.1–9.5)	8.5 (7.3–9.8)	7.8 (6.9–9.1)	** < 0.001**
Platelet (10^9^/L)	69 (48–110)	69 (49–105)	69 (47–113)	0.81
Total bilirubin (mg/dL)	3.4 (1.6–7.3)	2.0 (1.1–3.9)	5.0 (2.6–12.9)	** < 0.001**
Albumin (g/dL)	2.9 (2.6–3.3)	2.9 (2.8–3.3)	2.9 (2.4–3.1)	** < 0.001**
ALT (U/L)	35 (24–57)	34 (24–45)	41 (24–82)	**0.0026**
AST (U/L)	76 (46–155)	64 (43–112)	93 (53–262)	** < 0.001**
International normalized ratio	1.8 (1.5–2.3)	1.6 (1.4–1.8)	2.0 (1.7–2.7)	** < 0.001**
Prothrombin time (s)	19.4 (16.1–24.3)	17.2 (15.1–20.0)	22.1 (18.3–29.6)	** < 0.001**
Serum creatinine (mg/dL)	1.1 (0.8–1.9)	0.8 (0.7–1.0)	1.8 (1.1–2.8)	** < 0.001**
Blood urea nitrogen (mg/dL)	29.0 (18.0–48.0)	21.0 (14.0–33.3)	37.0 (23.0–58.0)	** < 0.001**
Serum sodium (mEq/L)	137 (133–140)	138 (135–141)	136(130–140)	** < 0.001**
Serum potassium (mEq/L)	4.5 (4.1–5.4)	4.4 (4.0–4.7)	4.8 (4.2–5.8)	** < 0.001**
Glucose (mg/dL)	125(109–158)	121(107–157)	129(112–160)	0.15
**Organ failures n (%)**				
Circulatory failure	94 (28.1)	9 (5.8)	85 (47.0)	** < 0.001**
Respiratory failure	105 (31.3)	2 (1.3)	103 (56.9)	** < 0.001**
Cerebral failure	94 (28.1)	16 (10.4)	78 (43.1)	** < 0.001**
Renal failure	96 (28.7)	0 (0)	96 (53.0)	** < 0.001**
Coagulation failure	64 (19.1)	2 (1.3)	62 (34.3)	** < 0.001**
Liver failure	54 (16.1)	4 (2.6)	50 (27.6)	** < 0.001**
**Specific treatments n (%)**				
Vasopressors	112 (33.4)	12 (7.8)	100 (55.2)	** < 0.001**
Mechanical ventilation	134 (40.0)	38 (24.7)	96 (53.0)	** < 0.001**
Renal replacement therapy	40 (11.9)	0 (0)	40 (22.1)	** < 0.001**
**Prognostic score at admission**				
CTP	11 ( 8–12)	8 (7–11)	12 (10–13)	** < 0.001**
MELD	20 ( 14–29)	14 (11–18)	28 (21–34)	** < 0.001**
MELD-Na	22 ( 14–34)	15 (12–19)	31 (23–40)	** < 0.001**
CTP class (A/B/C) n (%)	25(7.5)/95(28.4)/215(64.2)	24(15.6)/71(46.1)/59(38.3)	1(0.6%)/24(13.3)/156(86.2)	** < 0.001**
Length of ICU stay (days)	2 (1–4)	2 (1–3)	4 (2–6)	** < 0.001**
Length of hospital stay (days)	7 (4–16)	5 (4–9)	12 (5–21)	** < 0.001**
6-week mortality n (%)	92 (27.5)	13 (8.4)	79 (43.6)	** < 0.001**

Data were described as means (± standard deviation), medians (interquartile range) or numbers (percentage) where appropriate and compared with independent sample t-test, Mann–Whitney test and Chi square test accordingly

*SPO2* percutaneous oxygen saturation, *FIO2* fraction of inspired oxygen, *ALT* alanine aminotransferase, *AST* aspartate aminotransferase

### Cumulative survival rates of cirrhotic patients hospitalized for AVB

**Fig. 1:**
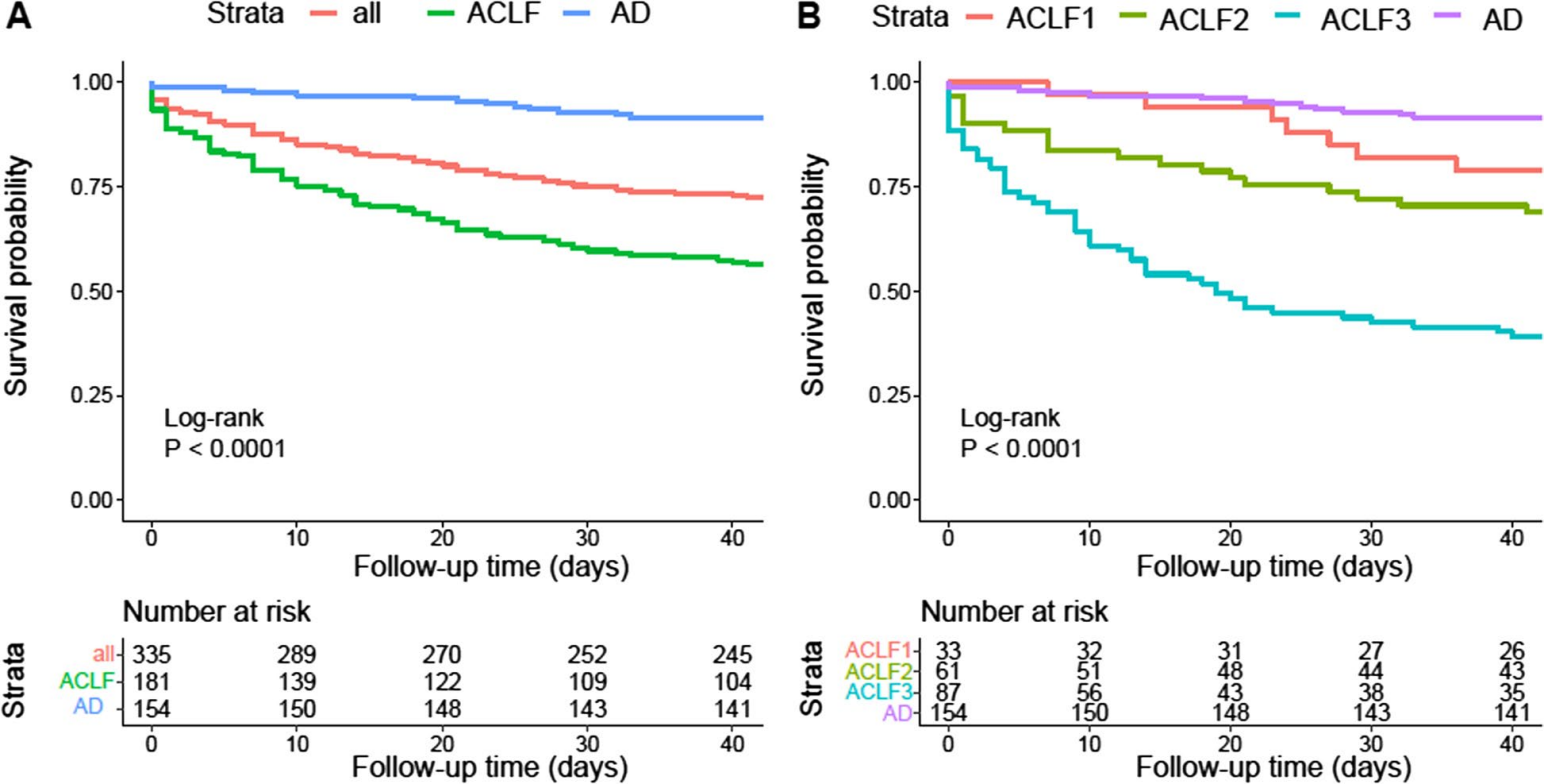
Cumulative survival rates of cirrhotic patients hospitalized for AVB. **A** The 42-day cumulative survival rate of ACLF patients was significantly lower than that of AD patients (56.4% vs. 91.6%, *P* < 0.001). **B** The 42-day cumulative survival rate of patients with ACLF gradually decreases with the increase of ACLF grade (*P* < 0.001)

Among the 335 included patients, 92 (27.5%) patients (ACLF = 79, AD = 13) died during a 42-day follow-up period. The cumulative 42-day survival rate in patients with ACLF was significantly lower than in those with AD (56.4% *vs.* 91.6%, *P* < 0.001) (Fig. 1A) and decreased gradually as the grade of ACLF increased (*P* < 0.001) (Fig. 1B). 22.5%, 34.2% and 63.8% of patients with ACLF grade 1, 2 and 3 died during the follow-up period, respectively (Fig. 1B).

### ACLF as an independent risk factor of 6-week mortality in cirrhotic patients with AVB

**Fig. 2:**
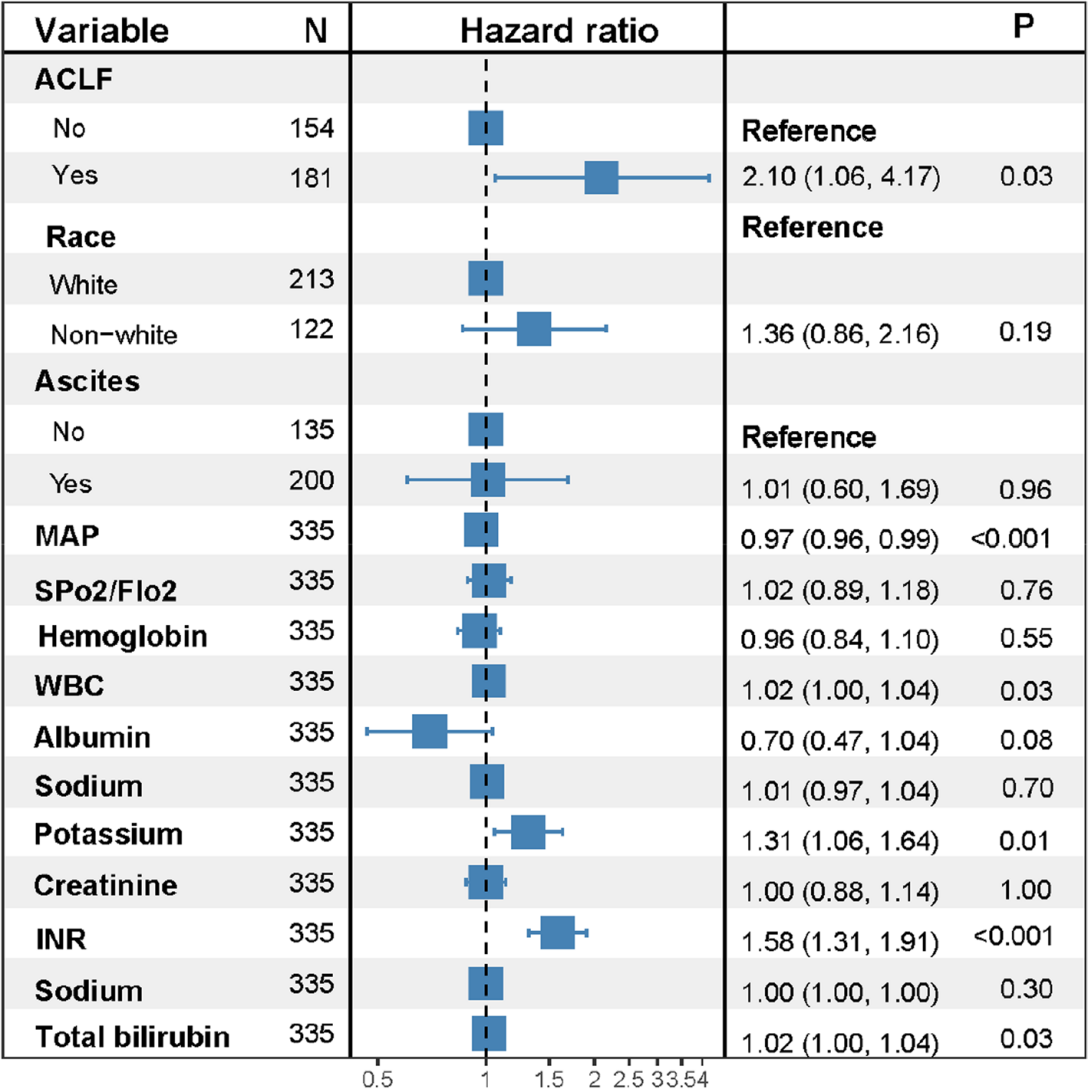
Forest plot showing the risk factors for 6-week mortality in cirrhotic patients with AVB. MAP, mean arterial pressure; SPO2, percutaneous oxygen saturation; FIO2, fraction of inspired oxygen

To further identify the role of ACLF in the prognosis of cirrhotic patients hospitalized for AVB, we performed Cox-proportional hazards regression analysis based on presence or absence of ACLF at admission and some well recognized risk factors for the prognosis of cirrhotic patients with AVB. In univariable analysis, variables with statistical differences included race, mean arterial pressure, SPO2 (percutaneous oxygen saturation)/FIO2 (fraction of inspired oxygen), presence of ACLF at admission, ascites, white blood cell, hemoglobin, albumin, total bilirubin, INR, ALT, serum sodium, serum potassium and serum creatinine (Table 2). Multivariable analysis showed that only ACLF (HR, 2.12, 95% CI: 1.07–4.20, *P* = 0.03), mean arterial pressure (HR, 0.97, 95% CI: 0.95–0.98, *P* < 0.001), white blood cell (HR, 1.02, 95% CI: 1.00–1.04, *P* = 0.03), total bilirubin (HR, 1.02, 95% CI: 1.00–1.04, *P* = 0.03), INR (HR, 1.58, 95% CI: 1.31–1.90, *P* < 0.001) and serum potassium (HR, 1.31, 95% CI: 1.05–1.63, *P* = 0.02) remained in the final regression model (Fig. 2).

**Table 2:** Risk factors for 6-week mortality in cirrhotic patients hospitalized for AVB

Variable	Univariable analysis		Multivariable analysis	
	HR (95% CI)	*P* value	HR (95% CI)	*P* value
**Age**	1.00 (0.99–1.02)	0.75		
Female	0.76 (0.48–1.20)	0.23		
Non-white race	1.79 (1.19–2.69)	**0.006**	1.36 (0.86–2.15)	0.19
Nonalcoholic cirrhosis	0.91 (0.59–1.41)	0.69		
ACLF	6.53 (3.63–11.75)	** < 0.001**	2.12 (1.07–4.20)	**0.03**
Infection	1.33 (0.86–2.05)	0.20		
Ascites	2.13 (1.33–3.42)	**0.002**	1.00 (0.60–1.67)	0.99
Portal vein thrombosis	1.57 (0.89–2.77)	0.12		
Mean arterial pressure	0.92 (0.89–0.94)	** < 0.001**	0.97 (0.95–0.98)	** < 0.001**
SPO2/FIO2	1.00 (1.00–1.00)	**0.022**	1.03 (0.89–1.18)	0.72
White blood cell	1.04 (1.03–1.05)	** < 0.001**	1.02 (1.00–1.04)	**0.03**
Hemoglobin	0.83 (0.73–0.94)	**0.003**	0.96 (0.84–1.10)	0.54
Platelet	1.00 (0.99–1.00)	0.09		
Total bilirubin	1.04 (1.03–1.05)	** < 0.001**	1.02 (1.00–1.04)	**0.03**
Albumin	0.37 (0.26–0.53)	** < 0.001**	0.69 (0.46–1.03)	0.07
Alanine aminotransferase	1.00 (1.00–1.00)	**0.007**	1.00 (1.00–1.00)	0.29
International normalized ratio	1.92 (1.67–2.20)	** < 0.001**	1.58 (1.31–1.90)	** < 0.001**
Serum creatinine	1.27 (1.17–1.38)	** < 0.001**	1.00 (0.88–1.13)	0.95
Serum sodium	0.96 (0.93–0.99)	**0.009**	1.01 (0.97–1.04)	0.72
Serum potassium	1.48 (1.23–1.79)	** < 0.001**	1.31 (1.05–1.63)	**0.02**
**Prognostic scores**				
CTP	1.38 (1.25–1.53)	** < 0.001**		
MELD	1.10 (1.08–1.13)	** < 0.001**		
MELD-Na	1.06 (1.05–1.08)	** < 0.001**		

*SPO2* percutaneous oxygen saturation, *FIO2* fraction of inspired oxygen

### Performances of prognostic scores

#### Discrimination

**Fig. 3:**
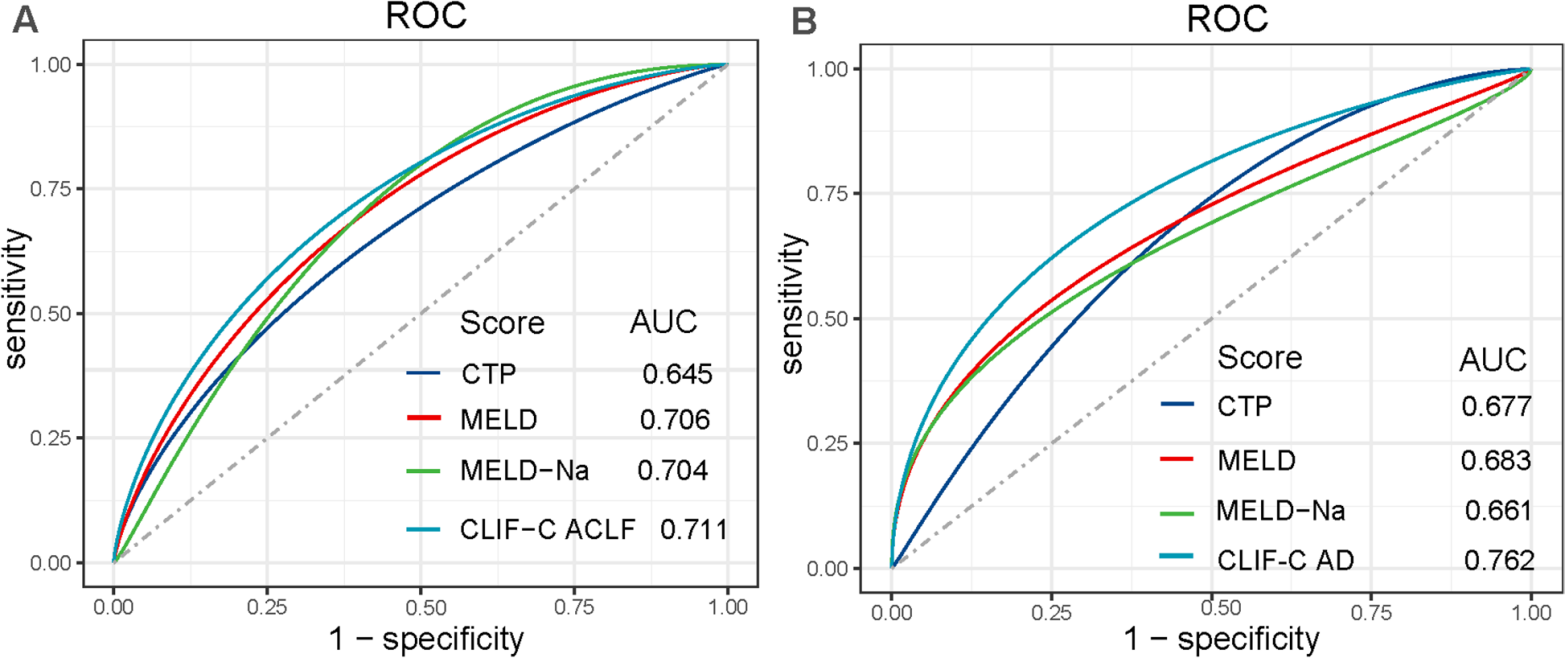
Discrimination performance of prognostic scores in ACLF **A** and AD patients **B**

In ACLF patients, the AUC regarding CTP, MELD, MELD-Na and CLIF-C ACLF were 0.645 (95% CI, 0.566–0.724), 0.706 (95% CI, 0.631–0.782), 0.704 (95% CI, 0.628–0.780) and 0.711 (95% CI, 0.636–0.786), respectively (Fig. 3A). No statistical differences between the AUC was found in DeLong test (*P* > 0.05 for all). The Youden index, cutoff value, sensitivity and specificity for above mentioned prognosis scores were 0.24/11/73.4/51.0, 0.34/25/77.2/56.9, 0.37/29/76.0/60.8 and 0.38/61/67.1/70.6, respectively (Table 3). In AD patients, the AUC regarding CTP, MELD, MELD-Na and CLIF-C AD were 0.677 (95% CI, 0.517–0.837), 0.683 (95% CI, 0.499–0.867), 0.661 (95% CI, 0.474–0.847) and 0.762 (95% CI, 0.615–0.909), respectively (Fig. 3B). In DeLong test, except MELD and MELD Na (*P* = 0.02), there was no statistical difference between the AUC (*P* > 0.05). The cutoff value, sensitivity and specificity for above mentioned prognosis scores were 10/61.5/76.6, 14/72.7/55.4, 23/40.9/88.1 and 55/77.3/68.4, respectively (Table 3).

**Table 3:** Predictive values of prognostic scores

Scores	AUC	Youden index	Cutoff value	SEN	SPE	PPV	NPV	Brier	R^2^	*P* in H–L test
**ACLF patients**										
CTP	0.645	0.24	11	73.4	51.0	53.7	71.2	0.229	0.092	0.16
MELD	0.706	0.34	25	77.2	56.9	58.1	76.3	0.215	0.166	0.79
MELD-Na	0.704	0.37	29	76.0	60.8	60.0	76.5	0.218	0.153	0.06
CLIF-C ACLF	0.711	0.38	61	67.1	70.6	63.9	73.5	0.211	0.195	0.52
**AD patients**										
CTP	0.677	0.38	10	61.5	76.6	19.5	95.6	0.075	0.063	0.45
MELD	0.683	0.40	23	46.2	94.3	42.9	95.0	0.072	0.101	0.77
MELD-Na	0.661	0.36	23	46.2	90.1	30.0	94.8	0.071	0.105	0.61
CLIF-C AD	0.762	0.46	55	76.9	69.5	18.9	97.0	0.067	0.190	0.73

*AUC* area under receiver operating characteristic curve, *SEN* sensitivity, *SPE* specificity, *PPV* positive predictive value, *NPV* negative predictive value, *H–L test* Hosmer–Lemeshow goodness-of-fit test

#### Calibration

**Fig. 4:**
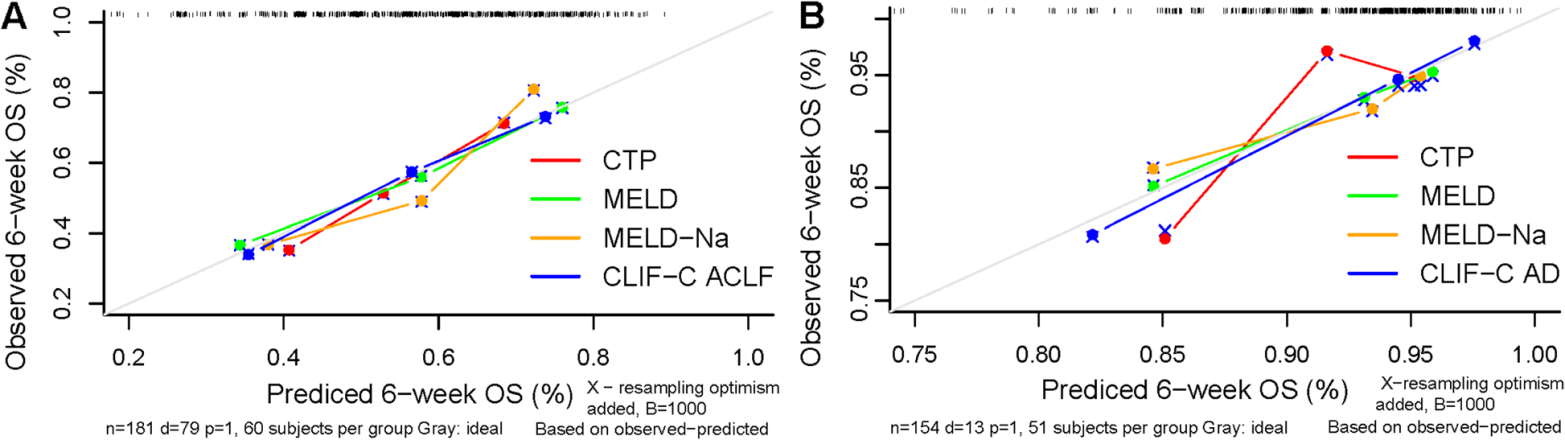
Calibration performance of prognostic scores in ACLF **A** and AD patients **B**

In ACLF patients, the *P* value in Hosmer–Lemeshow goodness-of-fit test for CTP, MELD, MELD-Na and CLIF-C ACLF were 0.024, 0.650, 0.004 and 0.491, respectively (Table 3); the concordance between observed and predicted 6-week mortality shown by CLIF-C ACLF was excellent and was superior to that shown by the other three prognostic scores (Fig. 4A).

In AD patients, the *P* value in Hosmer–Lemeshow goodness-of-fit test for CTP, MELD, MELD-Na and CLIF-C AD were 0.677, 0.683, 0.661 and 0.762, respectively (Table 3); the concordance between observed and predicted 6-week mortality shown by CLIF-C AD was excellent and comparable to that shown by MELD but superior to that shown by CTP and MELD-Na (Fig. 4B).

## Overall performance

In ACLF patients, the Brier score and R^2^ value for CTP, MELD, MELD-Na and CLIF-C ACLF were 0.229/0.104, 0.210/0.203, 0.213/0.187 and 0.209/0.219, respectively. In AD patients, the Brier score and R^2^ value for CTP, MELD, MELD-Na and CLIF-C AD were 0.094/0.077, 0.094/0.068, 0.093/0.073 and 0.087/0.173, respectively (Table 3).

## Discussion

Acute variceal bleeding (AVB) is a life-threatening complication of cirrhosis. Acute-on-chronic liver failure (ACLF) is a syndrome characterized by acute decompensation of cirrhosis, multiple organ failures and high short-term mortality. In this single center retrospective cohort study, we investigated the role of ACLF in the prognosis of cirrhotic patients with AVB and found that the cumulative 6-week survival rate in patients with ACLF was significantly lower than that in those without ACLF and as the ACLF grades increased, the 6-week survival rate significantly decreased. In addition, the presence of ACLF at admission remained as a risk factor for 6-week mortality in cirrhotic patients with AVB after adjusting for confounding factors. Compared with the traditional prognostic scores, CLIF-C ACLF and CLIF-C AD performed best in patients with and without ACLF, respectively. To the best of our knowledge, this was the first study to validate the prediction performance of prognostic scores in AVB patients with and without ACLF, respectively.

In the last decade, different definitions of ACLF have been developed by multiple international consortia [14, 26–28]. Among them, we choose the EASL-CLIF definition because it was developed entirely in cirrhotic patients with various acute decompensations and therefore most suitable for the design of our study. In this study, patients with ACLF more frequently presented with bacterial infections and had significantly higher peripheral blood WBC count, which is consistent with the current mainstream view that ACLF is closely associated with an intense systemic inflammation which may induce immune-mediated tissue damage and mitochondrial dysfunction contributing to the development of organ failures [29–32]. ACLF patients also more frequently presented with ascites, which is an independent predictive factor of kidney failure, the most common organ failure in ACLF [33, 34]. In addition, ACLF patients had significantly higher serum potassium and lower serum sodium; serum potassium was identified as one of the independent risk factors for 6-week mortality in AVB patients (HR, 1.31, *P* = 0.02). Hyperkalemia might present a more severe condition involving intensive systemic inflammation, renal failure, liver failure and sarcopenia [35]. Hyponatremia has been well described in associations with hepatorenal syndrome, ascites and liver-related mortality [11]. Briefly speaking, our study conducted in AVB patients supports the view that ACLF is a more severe entity than AD [36], and it is very important to identify patients with ACLF at the early stage because it can increase their chances of receiving organ support treatment and/or intensive care, and correspondingly reduce their mortality.

Although it is well recognized that cirrhotic patients with ACLF carry a high short-term mortality, there are limited literature on the role of ACLF in the prognosis of cirrhotic patients with AVB, exclusively [37–39]. In this study, the 6-week mortality in AVB patients with ACLF was significantly higher than those without ACLF (43.6% *vs.* 8.4%, *P* < 0.0001) and increased significantly as the grade of ACLF increased (grade 1: 22.5%, grade 2: 34.2% and grade 3: 63.8%, *P* < 0.0001). The overall severity of ACLF is consistent with the result in CANONIC study (28-day mortality for AD, ACLF, ACLF grade 1, grade 2 and grade 3: 4.7%, 33.9%, 22.1%, 32.0% and 76.7%) and some recent studies on AVB by Shin et al. (28-day mortality AD:3.4%, ACLF:41.0%, grade 1: 7.1%, grade 2: 28.6% and grade 3: 80.8%) [37], Trebicka et al. (42-day mortality AD:10.0%, ACLF:47.1%, grade 1: 30.0%, grade 2: 50.0% and grade 3: 70.0%) [38] and Kumar et al. (42-day mortality AD:9.1%, ACLF:47.9%, grade 1: 24.0%, grade 2: 44.0% and grade 3: 77.0%) [39]. The different mortality in AVB patients with ACLF between our study and previous studies might be due to the different baseline characteristics of included patients. For example, Shin’s study included patients hospitalized either in ICU or general ward, Trebicka’s study included patients with HCC and Kumar’s study only included patients with refractory AVB, probably resulting in a relatively lower or higher mortality than our study. In addition, presence of ACLF at admission increased a 1.12 fold risk of death within 6-week after adjusting for confounding factors (HR = 2.12, *P* = 0.03), which was consistent with the result in study by Trebicka et al. (HR = 2.72, *P* < 0.001). In short, the 6-week mortality in AVB patients with ACLF was significantly higher than those with mere AD; ACLF is an independent risk factor for the 6-week mortality in cirrhotic patients complicated with AVB.

So far, few studies exclusively validated the performance of prognostic scores in AVB patients with or without ACLF. In this study, the AUC value of CLIF-C ACLF for 6-week mortality in AVB patients with ACLF is higher than the traditional scores, such as CTP, MELD and MELD-Na (although without statistic difference); the calibration ability of CLIF-C ACLF is excellent and superior to the traditional scores. In AD patients, CLIF-C AD was the only prognostic score with AUC > 0.7; the calibration ability of CLIF-C AD is also excellent and comparable to that of MELD but superior to that of CTP and MELD-Na. This is consistent with the result in our previous study in which CLIF-C AD outperformed the traditional prognostic scores in the prediction of 6-week mortality of AVB patients hospitalized in the general ward [40]. The superior performance of CLIF-C ACLF and CLIF-C AD to traditional prognostic scores might be due to the distinct background where these prognostic scores were developed. CTP and MELD were developed in cirrhotic patients who received surgery and TIPS therapy due to recurrent esophagogastric variceal bleeding and various complications of portal hypertension, respectively. MELD-Na was developed in cirrhotic patients listed for liver transplantation. All these three prognostic scores were developed regardless of the presence of ACLF. On the other hand, CLIF-C ACLF and CLIF-C AD were developed in cirrhotic patients included in the CANONIC study with and without ACLF, respectively. Thus, a superior performance of these two prognostic scores could be expected. According to the cutoff values of CLIF-C ACLF and CLIF-C AD, ACLF patients with CLIF-C ACLF score > 61 and AD patients with CLIF-C AD score > 55 may need to be stratified as high risk, respectively. These high risk patients may need to be provided with organ support therapy in intensive care unit [21] or salvage treatment such as pre-emptive TIPS [38] or Rescue TIPS [39], which were recently found to be very effective to reduce the 6-week and 1 year mortality in AVB patients with ACLF.

Our study has some limitations. First, as this was a single center and observational study, selection, information and confounding biases were inevitable. Second, although the medical record information in MIMIC database was prospectively and timely collected, the diagnosis of covert hepatic encephalopathy (minimal hepatic encephalopathy and grade I) might be partly effected by the subjective factors of observers. Besides, the diagnosis of respiratory failure or brain failure became difficult when the mechanical ventilation was provided because it was hard to determine the exact reason for the mechanical ventilation therapy (respiratory failure, airway protection in brain failure or both), which might to some extent lead to a bias in the diagnosis of respiratory failure or brain failure. Nonetheless, since the mortality in our cohort was consistent with that described in recent prospective studies on ACLF [37–39], we believe that our results are highly credible. Finally, as MIMIC database only comprises medical data of patients hospitalized in ICU, we did not validate the performance of prognostic scores in AVB patients hospitalized in the general ward.

In conclusion, ACLF at admission is an independent predictor for the 6-week mortality in cirrhotic patients with AVB. To improve the prognosis of this entity, it is essential to make treatment plans according to the presence or absence of ACLF at admission. CLIF-C ACLF and CLIF-C AD outperformed other prognostic scores in the prediction of 6-week mortality of patients with and without ACLF, respectively, and can be used for the risk stratification of these two distinct entities.

## Data Availability

Data in this study are available from https://www.scidb.cn/anonymous/SWI2Ymky.

## Abbreviations

AVB: Acute variceal bleeding
ACLF: Acute-on-chronic liver failure
AUC: Area under the curve
CLIF-CACLF: Chronic liver failure-organ failure-Consortium acute-on-chronic liver failure
CLIF-C AD: Chronic liver failure-organ failure-Consortium acute decompensation
CLIF-OF: Chronic liver failure-organ failure
FIO2: Fraction of inspired oxygen
HE: Hepatic encephalopathy
H-L test: Hosmer-Lemeshow goodness-of-fit test
MAP: Mean arterial pressure
PVT: Portal vein thrombosis
SPO2: Percutaneous oxygen saturation
SBP: spontaneous bacterial peritonitis
